# Retinoblastoma protein expression is an independent predictor of both radiation response and survival in muscle-invasive bladder cancer

**DOI:** 10.1038/sj.bjc.6601063

**Published:** 2003-07-15

**Authors:** M Agerbaek, J Alsner, N Marcussen, F Lundbeck, H von der Maase

**Affiliations:** 1 Department of Oncology, Aarhus University Hospital, Norrebrogade 44, DK8000 Aarhus C, Denmark; 2 Department of Experimental Clinical Oncology, Aarhus University Hospital, Norrebrogade 44, DK8000 Aarhus C, Denmark; 3 Department of Pathology, Aarhus University Hospital, Norrebrogade 44, DK8000 Aarhus C, Denmark; 4 Department of Urology, Aarhus University Hospital, Brendstrupgaardsvej, DK8200 Aarthus N, Denmark

**Keywords:** retinoblastoma protein, bladder cancer, radiotherapy, prognosis

## Abstract

The objective of the study was to investigate the predictive value of various clinical, biochemical, and histopathological parameters, with special emphasis on the expression of the retinoblastoma protein (pRB), on the radiation response in bladder cancer. In order to obtain a truly objective response measure, patients receiving preoperative radiotherapy followed by cystectomy were studied. Pretreatment tumour samples and clinical data from 108 consecutive patients were collected. End points were complete response (CR) to radiotherapy, relapse-free survival time and overall survival time. Expression of pRB was assessed by immunohistochemical staining as present or absent. Complete response to radiotherapy was obtained in 42 of 106 evaluable patients (40%). Predictive for CR to radiotherapy, in univariate analysis, was transurethral resection (as opposed to biopsy), B-haemoglobin, no upper urinary retention, and loss of pRB staining. Loss of pRB staining was the strongest independent predictor of radiation response in multivariate logistic regression analysis and absence of upper urinary retention was the only other significant factor. Loss of pRB was the only parameter showing statistically significant, independent association with relapse-free survival, whereas B-haemoglobin was also independently associated with overall survival. Loss of pRB expression seems to indicate a phenotype displaying enhanced radiosensivity and may be of benefit by denoting patients who would selectively benefit from a treatment schedule containing radiotherapy.

The role of radiotherapy in the treatment of muscle-invasive bladder cancer is changing. Although the superiority of radical cystectomy over curatively intended radiotherapy has never been tested in randomised trials, retrospective analyses seem to favour the first (Shelley *et al*, 2001). For the vast majority of patients cystectomy is, consequently, the treatment of choice in most countries. Definite radiotherapy is offered to patients medically unfit for major surgery, patients refusing cystectomy, and patients with locally advanced disease (T4 or N1 disease) in most centres. Lasting local control can, however, be achieved by radiotherapy in a significant proportion of patients.

Encouraged by the obvious benefits of bladder conservation in terms of quality of life, multimodality bladder conserving therapies, combining organ sparing surgery with chemo-and radiotherapy, have been designed and promising results have been published (Dunst *et al*, 1994; Kaufman *et al*, 2000; Kim and Steinberg, 2000; Rodel *et al*, 2002). Radiotherapy, therefore, seems to be re-entering the treatment of bladder cancer. However, organ-sparing treatment is an option only in select patients, reflecting the fact that much is to be gained by improving patient selection or individualising treatment of the specific patient. Our tools for this task are limited as only TNM stage, grade, and performance status are widely recognised as decisive for the choice of treatment.

Tumour heterogeneity in terms of radiosensivity in bladder cancer is remarkable (Barnetson *et al*, 1999), and only to a limited extent correlated to conventional clinical and histopathological parameters. The rapidly increasing insight into the molecular factors responsible for malignant transformation, cell cycle control, and response to therapy offers new, potentially prognostic, information on individual tumours. The retinoblastoma protein (pRB) is both a tumour suppressor and a pivotal player in the cells protection against genotoxic stress mainly by inducing arrest at the cell cycle checkpoints to allow DNA repair (Bartek and Lukas, 2001). Loss of expression of the *RB* gene both contributes to the oncogenesis *per se* and thwarts the ability of the tumour cell to respond to the toxic effects of radiotherapy. pRB-deficient cells have accordingly been found to be hypersensitive to DNA damage-induced apoptosis (Knudsen *et al*, 2000; Wang *et al*, 2001). In bladder cancer, pRB-negative tumours have been shown to have higher proliferative indices (Lipponen and Liukkonen, 1995), a quality that might well influence both radiosensitivity and clinical behaviour. Loss of pRB expression is found in a significant proportion of transitional cell bladder cancers, which supports its candidacy as a clinically relevant molecular marker of increased radioresponsiveness in this disease.

## MATERIALS AND METHODS

### Patients

A total of 108 consecutive patients with invasive transitional cell bladder cancer (T1–T4a) receiving preoperative radiotherapy followed by radical cystectomy at the Departments of Oncology and Urology, Aarhus University Hospital, in the period from 1980 to 1992 were included in the study. Clinical data of relevance were extracted from patient files. Paraffin-embedded pretreatment tumour biopsies and embedded sections of the excised bladder after radiotherapy were available from all patients. Tumour stage was determined by cystoscopy, biopsy or resection of the tumour and bimanual palpation under general anaesthesia in accordance with the UICC/AJCC recommendations (1997). Metastatic disease was excluded by chest X-ray, bone scan in the case of bone pain, ultrasonography of the upper abdomen in the case of abnormal liver biochemistry and, in the majority of the patients (100 pts.), by a CT scan of the pelvis. Transurethral resection of the bladder tumour (TUR-B) was performed prior to radiotherapy in 60 patients (56%), leaving macroscopic tumour tissue in the bladder wall in 44 (41%), no macroscopic tumour but residual tumour demonstrable in biopsies from the resected area in 14 (13%), and neither visible nor histopathologically demonstrable residual tumour in two patients (2%). In the remaining 48 patients, only biopsing of the bladder tumours was performed. A total of 106 of the 108 patients thus had verified residual tumour at the initiation of radiotherapy. Clinical data, pretreatment laboratory results, and pathology reports were retrieved from archives. Presence or absence of upper urinary retention was documented in 103 patients (95%) by intravenous pyelography and/or renography. Revision of the histological specimens was performed (by MA and NM) ensuring a uniform classification of pre-and post-treatment tumour stage and grade according to Bergkvist (1965). Presence or absence of CIS was recorded only in patients where systematic biopsing of bladder mucosa had been performed. It was noted whether patients were diagnosed with muscle-invasive disease on initial presentation or had experienced disease progression from a ‘pre-muscle-invasive stage’ (Ta, Tis, or T1) over a period of time (>3 months), and by that went through a period of surveillance allowing ‘early’ treatment of the muscle-invasive tumour.

### Radiotherapy

As treatment policies changed slightly over time, all patients included were treated according to one of three schemes: 40 Gy in 20 fractions (47 pt.),. 42 Gy in 20 fractions (50 pt.), or 46 Gy in 23 fractions (12 pt.); in all cases with five fractions per week. All but one patient received the planned radiation schedule; the last patient received 38 of planned 40 Gy, the last fraction being omitted for reasons not recorded. Radiotherapy was planned based on a CT scan in 100 patients, whereas the remaining eight were planned after conventional X-ray simulation. All treatments were given with a three-field box technique (anterior and two opposing lateral fields), using 8 MV photons for the anterior field and 16 MV for the lateral fields. Treatment volumes averaged 2210 cm^3^ (1260–4788; sd=632).

Response to radiotherapy was evaluated from the excised bladders as either complete response (CR)–absence of residual malignant cells in the cystectomy specimen upon thorough histopathological examination–, or incomplete response (IR)–presence of residual malignancy in cystectomy specimen. No attempt was made on evaluating partial responses/downstaging.

### Treatment of relapse

Of the 58 patients experiencing a relapse of bladder cancer after cystectomy, only three received potentially life-prolonging treatment with systemic chemotherapy. A total of 14 patients received palliative radiotherapy (mainly on metastatic sites) and three patients had palliative surgical resections. In all, 39 patients received only supportive treatment for their relapse.

### Immunohistochemical staining

The 4 cm sections of archival, formalin-fixed, paraffin-embedded tumour tissue were placed on silane-coated slides, deparaffinised in xylene, and rehydrated through graded alcohols. Endogenous peroxidase was quenched by rinsing with 0.5% hydrogen peroxide in absolute ethanol for 20 min followed by rinsing in Tris/PBS (pH 7.6) and water. Heat-induced antigen retrieval was performed using a T-EG buffer (Tris 10 mM+EGTA 0.5 mM; pH 9.0) and microwave heating for 3 × 5 min followed by cooling for 20 min in the buffer. After rinsing twice in Tris/PBS, slides were incubated overnight at 4°C with primary antibody (monoclonal mouse anti-human retinoblastoma gene product Rb1 (formerly designated 1F8), DAKO) diluted 1 : 200 in Antibody Diluent (DAKO). Following another two rinses in Tris/PBS, bound primary antibody was visualised by incubation with EnVision™+/HRP Mouse (DAKO) for 30 min, three rinses in Tris/PBS and 5 min in liquid DAB+ (DAKO). Sections were counterstained with Mayer's haematoxylin and mounted in dibutyl phthalate xylene medium (DPX, BDH Laboratory Supplies, WNR International, Poole, England).

Staining of nuclei of fibroblasts, endothelial cells, or other normal tissue components of the surrounding stroma served as individual positive controls of the staining reaction in each slide. Negative controls were performed for each batch by omitting the primary antibody. Tumours were classified as pRB negative when no staining of tumour cell nuclei was seen in sections showing positive staining of adjacent stromal cell nuclei. Cytoplasmic staining was disregarded. Six of the pRB-positive tumours contained areas of negatively staining tumour cells (mixed staining pattern). These were classified as pRB positive.

### Statistics

Categorical data were compared in univariate analysis using the *χ*^2^ test or Fischer's exact test where appropriate, and censored data were compared using log-rank test. Continuous variables were dichotomised using median value (age) or ‘normal limits’ (B-haemoglobin (above 8.0 mmol l^−1^ in males, above 7.0 mmol l^−1^ in females) and S-creatinine (below 110 μmol l^−1^)) as cutoff points. Multivariate analyses were performed using logistic regression analysis (response to radiotherapy) and Cox proportional hazards models (disease-free-and overall survival). All variables were tested in univariate analyses (*χ*^2^/Fischer's and log-rank test), and variables showing significant correlations with end points or variables indicated as prognostic factors in the literature were entered into the multivariate analyses. All statistical analyses were performed using the SPSS 10.0 statistical package (SPSS Inc., IL, USA).

## RESULTS

Complete response to preoperative radiotherapy was found in 42 of 106 patients (40%) with residual tumour after TUR-B. A strong correlation between radiation response and relapse (*P* < 0.001), relapse-free survival time (*P*<0.0001), and overall survival time (*P*=0.0001) was found (Table 1

**Table 1:** Correlations between complete response (CR) to radiotherapy and outcome in 106 evaluable patients (two patients not evaluable for response to radiotherapy because of microscopically radical TUR-B; n.r.: not reached)

	**Relapse (%)**	**Median relapse-free survival in months (s.e.)**	**Median overall survival in months (s.e.)**
CR (*n*=42)	13 (31)	n.r.	104 (74)
IR (*n*=64)	42 (68)	17 (2)	15 (3)
*P*-value	0.001	<0.0001	0.0001

). Table 2

**Table 2:** Patient characteristics and relation to outcome of radiotherapy in 106 evaluable patients

**Parameter**	**Number of patients**	**% CR to radiotherapy**	**% IR to radiotherapy**	***P*-value**
Age	56	*39*	*61*	
Above median	50	*40*	*60*	1.00
Below median				
				
Sex	89	*39*	*61*	
Male	17	*41*	*59*	1.00
Female				
				
Grade	1	*0*	*100*	
II	83	*40*	*60*	
III	22	*41*	*59*	0.72
IV				
				
Stage	4	*25*	*75*	
T1	27	*41*	*59*	
T2	29	*48*	*52*	
T3a	34	*38*	*62*	
T3b	12	*25*	*75*	0.67
T4a				
				
IVP or renography	74	*51*	*49*	
Normal	27	*15*	*85*	0.001
Upper urinary retention	5			
Not available				
				
S-creatinine	85	*44*	*56*	
Within normal limits	21	*24*	*76*	0.14
Above normal limits				
				
B-haemoglobin	78	*46*	*54*	
Within normal limits	28	*21*	*79*	0.03
Below normal limits				
				
CIS	36	*42*	*58*	
Yes	38	*37*	*63*	0.81
No	32			
Not available				
				
Pre-muscle-invasive stage	38	*37*	*63*	
Yes	68	*41*	*59*	0.69
No				
				
Radicality of TURB	48	*27*	*73*	
Biopsy	58	*50*	*50*	0.02
Resection				
Macroscopic complete TUR-B	14	*57*	*43*	
Macroscopic incomplete TUR-B	44	*48*	*52*	0.24
				
Histology	88	*41*	*59*	
Pure TCC	18	*33*	*67*	0.61
TCC with squamous/adenocarc. diff.				
Lymphovascular invasion	25	*40*	*60*	
No lymphovascular invasion	81	*39*	*61*	1.00
				
Papillary (partly or completely)	53	*43*	*57*	
Solid (completely)	53	*36*	*64*	0.55
				
Multifocality	14	*36*	*64*	
Yes	92	*40*	*60*	1.00
No				
				
Radiotherapy	12	*42*	*58*	
46 Gy	48	*33*	*67*	
42 Gy	46	*41*	*59*	0.28
40 Gy				
				
pRB	66	*29*	*71*	
Positive	40	*58*	*42*	0.004
Negative				

Two patients not evaluable for response to radiotherapy because of microscopically radical TUR-B; TCC=transitiocellular carcinoma, TUR-B=Transurethral resection of bladder tumour, IVP=intravenous pyelography, CIS=presence of carcinoma *in situ* in biopsies from bladder mucosa).

shows the various parameters examined, their distribution in the group of complete responders *v*s incomplete responders to radiotherapy, and the results of univariate analyses. No association between outcome of radiotherapy and age, sex, grade of atypia, histological type, pattern of growth, multifocality, lymphovascular invasion, coexistence of CIS, diagnose of a pre-muscle-invasive stage, or radiation dose was found. T stage was not significantly associated with the radiation response, where T3a tumours exhibited the highest proportion of complete responders (14 of 29=48%). Serum creatinine within normal limits and no macroscopic tumour left after TUR-B was more frequent among complete responders albeit not reaching statistical significance (*P*=0.14 and 0.24). Predictive for CR to radiotherapy in univariate analysis was resection at TUR-B (as opposed to biopsy) (*P*=0.02), B-haemoglobin within normal limits (*P*=0.03), no upper urinary retention on IVP or renography (*P*=0.001), and loss of pRB staining (*P*=0.004).

No correlation between pRB status and tumour grade or stage (or any other of the examined parameters) was seen. Significant correlations were noted between upper urinary retention and T-stage (*P*=0.03) and S-creatinine above normal limits (*P*=0.001) and between T stage and grade of atypia (*P*=0.008) (data not shown).

In multivariate logistic regression analysis, only absence of pRB immunostaining (*P*=0.004) and absence of upper urinary retention (*P*=0.032) were statistically significant independent predictors of CR to radiotherapy (Table 3

**Table 3:** Results of multivariate logistic regression analysis in 101 evaluable patients

**Parameter**	***P***	**OR**	**95% CI**
Negative pRB staining	0.004	4.07	1.57–10.53
Upper urinary retention	0.032	0.25	0.07–0.88
Biopsy only at TUR-B	0.094	0.46	0.18–1.15
B-haemoglobin below normal	0.212	0.45	0.13–1.58

Two patients not evaluable for response to radiotherapy because of microscopically radical TUR-B, five patients excluded because of lack of information on result of IVP or renography. Dependent variable: CR to radiotherapy. OR=overall response; CI=confidense interval.

).

**Figure 1:**
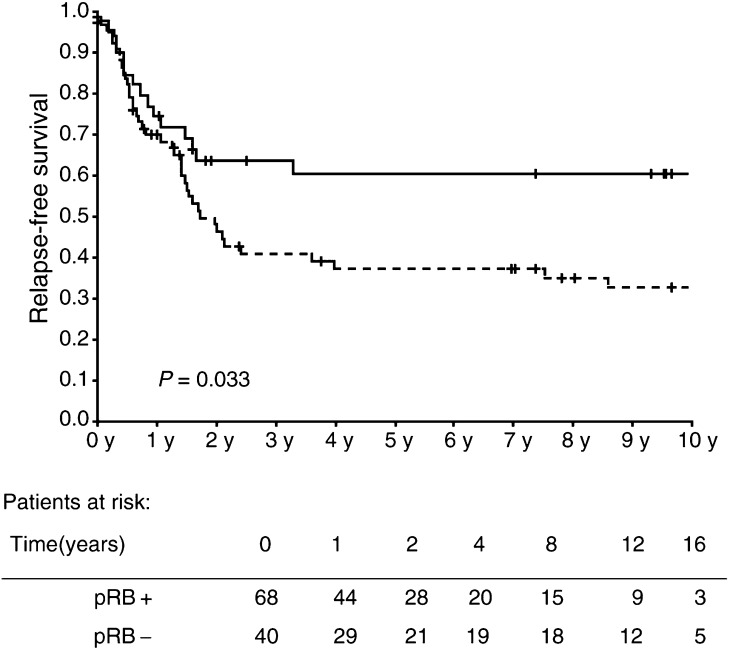
Kaplan–Meier plot of relapse-free survival of patients with pRB-negative (—) and pRB-positive (- -) tumours. (+) marks censoring events.

When tested individually for association with relapse-free survival time (using Kaplan–Meier/log-rank test, Figure 1

), only absence of pRB staining showed statistical significance as a prognostic marker (*P*=0.033). T stage was not significantly associated with survival but showed the most marked separation between survival of groups when divided in to two: ‘T3b and higher’ and ‘T3a and lower’, where a P-value of 0.22 was seen. Grade was significantly associated with disease-free survival after 1 year (*P*=0.018), but not with further follow-up (P=0.299) (Figure 3). Separation, although not statistically significant, was also seen for biopsy *vs* resection at TUR-B (*P*=0.07) and B-haemoglobin within or below normal limits (*P*=0.17). When entering the above factors in a Cox regression analysis (Table 4

**Table 4:** Results of Cox regression analysis including all 108 patients. Dependent variable: relapse-free survival

Parameter	P	OR	95% CI
Negative pRB staining	0.030	0.51	0.28–0.94
B-haemoglobin below normal	0.170	1.52	0.84–2.77
Biopsy only at TUR-B	0.144	1.48	0.87–2.52
Grade 4	0.314	1.39	0.73–2.65
T stage3B	0.591	1.16	0.68–1.96

), only pRB had statistically significant independent prognostic significance (*P*=0.030). Changing the end point to overall survival time made B-haemoglobin a significant prognostic marker along with pRB (Figure 2

**Figure 2:**
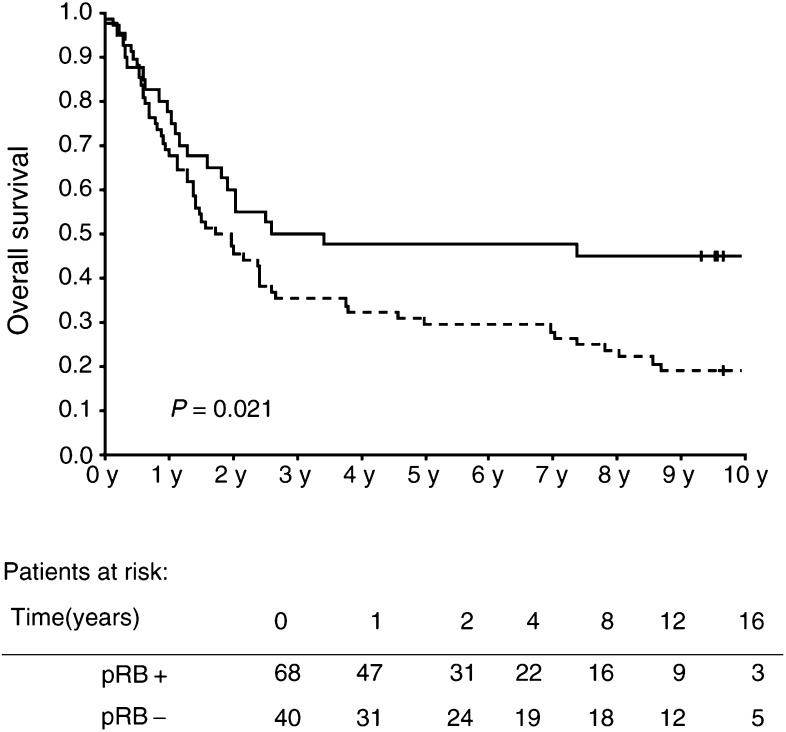
Kaplan–Meier plot of overall survival of patients with pRB-negative (—) and pRB-positive (- -) tumours. (+) marks censoring events.

and Table 5

**Table 5:** Results of Cox regression analysis including all 108 patients. Dependent variable: overall survival

**Parameter**	***P***	**OR**	**95% CI**
Negative pRB staining	0.009	0.52	0.32–0.85
B-haemoglobin below normal	0.002	2.13	1.31–3.44
Grade 4	0.111	1.54	0.91–2.61
Biopsy only at TUR-B	0.294	1.27	0.81–1.99
T stage⩾3B	0.868	1.04	0.66–1.64

) in univariate analysis (*P*=0.003) as well as in multivariate Cox regression (*P*=0.002) and the statistical significance of pRB status was strengthened in both (*P*=0.021 and 0.009, respectively).

## DISCUSSION

Apart from T staging, classical clinical and histopathological parameters have shown limited value as predictors for the radiocurability of muscle-invasive bladder cancer. The present study supports this view, as neither grade of anaplasia nor histological pattern of growth and differentiation, lymphovascular invasion, multifocality, simultaneous presence of CIS, or the registration of a pre-muscle-invasive stage correlated significantly with the tumour response to radiotherapy. T stage has previously been demonstrated to be predictive for the outcome in bladder cancer subjected to radical radiotherapy (Sengelov and von der Maase, 1999). However, when cohorts receiving preoperative radiotherapy have been examined, correlations have been less consistent (Pollack *et al*, 1997b; Rotterud *et al*, 2001). In this context, it is important to realise that the supposed benefit from preoperative radiotherapy lies in an effect on micrometastatic spread at the time of surgery and not in the eradication of the primary tumour. Complete response to radiotherapy is thus an indicator of radiosensitivity, but not a prerequisite for the desired end point. Undoubtedly, the low precision of clinical staging documented in many surgical series (Pagano *et al*, 1991; Cheng *et al*, 2000) contributes greatly to the lack of predictive value of T staging. The recognition of upper urinary retention as a prognostic marker for radiosensitivity *per se* probably reflects the ability of this examination to reveal an advanced local tumour stage as reflected by the observed significant association between T stage and upper urinary retention, rather than the effect of impaired renal function.

**Figure 3:**
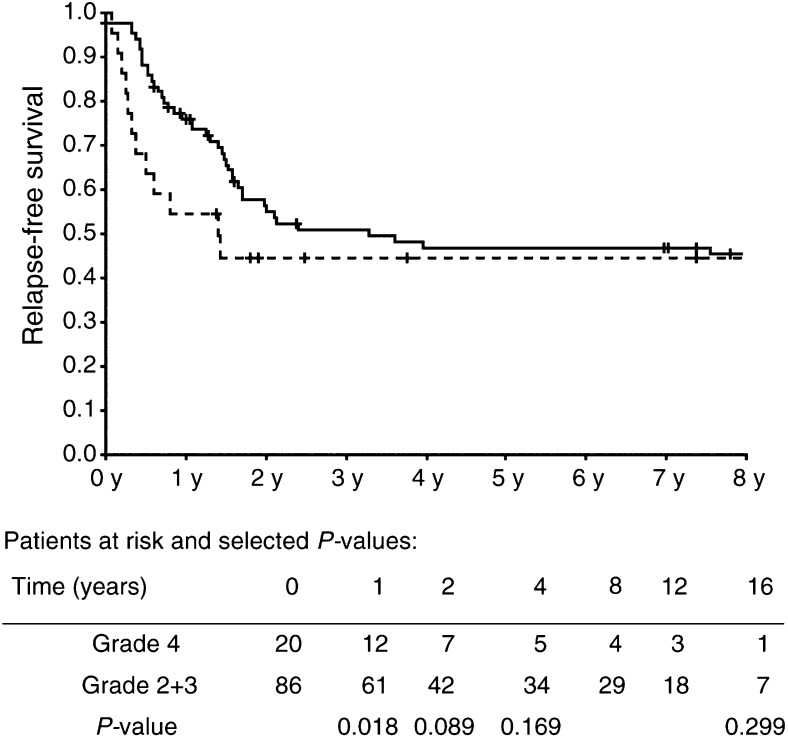
Kaplan–Meier plot of relapse-free survival of patients with grade 2+3 (—) and grade 4 (- -) tumours. (+) marks censoring events. *P* values calculated with 1, 2, 4, and 16 years of follow-up.

The effects of subnormal haemoglobin concentration could be dual. Low haemoglobin increases the risk of tumour hypoxia, which has been demonstrated to influence the outcome of radiotherapy in bladder cancer (Gospodarowicz *et al*, 1989; Hoskin *et al*, 1999). Anaemia, on the other hand, also often accompanies advanced cancer disease as well as other wasting diseases and may, consequently, be a marker hereof and as such associated with decreased overall survival. In the present study, B-haemoglobin below normal limits was significantly correlated to IR in univariate analysis, suggesting an effect of hypoxia. The statistical significance, however, disappeared in the multivariate analysis. B-haemoglobin below normal limits was independently associated with decreased overall survival but not with disease-free survival in the Cox analysis, implicating a more general effect. Grade of atypia of the tumour has been shown to carry independent prognostic information concerning survival in some studies of radical radiotherapy in bladder cancer (Gospodarowicz *et al*, 1989; Greven *et al*, 1990), while others have failed to find such an association (Fossa *et al*, 1993; Fung *et al*, 1991). The present study did not show statistically significant correlation between grade and CR to radiotherapy. It was, however, noted (Figure 3

) that grade 4 tumours relapsed earlier, although not more frequently,- than tumours of lower grades (grade 3), resulting in a significant difference in relapse-free survival after 1 year of follow-up. After 2 years, the difference was reduced to a statistically insignificant level (*P*=0.089) and survival curves merged beyond 4 years of follow-up.

The extent of resection obtained at TUR-B reflects both the size and extent of the tumour present and the treatment policy of the surgeon. No systematic information regarding the size of the tumours was available, so a possible association with this parameter could not be tested. The surgical procedure preceding radiotherapy (incomplete resection *vs* biopsy) correlated significantly with the response to treatment, as would be expected. The association, however, was not independent, as it was not statistically significant in the multivariate analysis. Macroscopic complete resection was obtained in 14 patients, but this was, surprisingly, not significantly associated with CR or survival. This could in part be ascribable to the low number of patients in whom complete resection was obtained, but could also be an indicator that the combined clinical and histopathological diagnose of complete resection is not a good reflection of the actual situation.

The strongest and most consistent independent predictor of both CR to radiotherapy and survival was found to be the expression of pRB. Loss of expression of pRB was observed in 37% of tumours (40 of 108), a number corresponding well with the findings of other studies (Geradts *et al*, 1994; Cordon-Cardo *et al*, 1997; Pollack *et al*, 1997a, 1997b; Cote *et al*, 1998; Moonen *et al*, 2001). Thus, loss of this cell cycle control factor is seen in a clinically relevant proportion of bladder tumours adding to its relevance as a predictive factor for radiosensitivity. Two published series of bladder cancers, applying radically intended radiotherapy, have investigated the effects of pRB expression on the outcome (Jahnson *et al*, 1995; Moonen *et al*, 2001). The study by Jahnson *et al* (1995) included 154 patients and showed more complete tumour responses (defined as absence of tumour at cystoscopy with biopsies at 6–7 months) and increased cancer–specific survival among pRB-negative patients, although without reaching statistical significance. The recent study by Moonen *et al* (2001) evaluated 83 patients with a relatively high proportion of early-stage tumours (39% T1, 45% T2, 16% T3, and no T4) treated with a wide range of total doses (46–72 Gy) and doses per fraction (1.8–3 Gy, mean 2.4 Gy). This study also found loss of pRB staining to be associated with increased local control (70% *vs* 39% free of local recurrence at 3 years), but again without reaching statistical significance in either uni-or multivariate analysis. Besides our investigation, another study has employed the results of preoperative radiotherapy for evaluating the radioresponsiveness of bladder cancer in relation to pRB. The study by Pollack *et al* (1997a) found pRB status to be the only pretreatment prognostic factor that was independently associated with complete radiation response, but could only demonstrate a survival benefit in a subgroup analysis for stage T3B patients. In striking contrast to these findings, when pRB status has been studied in a series of purely surgically managed bladder cancer, loss of pRB expression has consistently been related to aggressive disease course and decreased survival (Cordon-Cardo *et al*, 1992; Logothetis *et al*, 1992; Cordon-Cardo *et al*, 1997; Cote *et al*, 1998; Grossman *et al*, 1998).

In conclusion, the results of the present investigation demonstrate pRB immunoreactivity as an independent predictive factor for radiosensitivity and an independent prognostic factor for survival in muscle-invasive bladder cancer. The available literature seems to indicate that loss of RB protein expression in bladder cancer confers a more aggressive phenotype resulting in decreased survival when applying an exclusively surgical strategy but also an increased radiosensitivity resulting in at least equal results in radically irradiated series and in a survival benefit when modalities are combined. It follows that treatment of pRB-negative bladder cancers might selectively benefit from radiotherapy either alone or as preoperative radiotherapy followed by cystectomy, or perhaps as part of the newer bladder sparing regimens with aggressive local surgery and combined radiotherapy and chemotherapy.
